# A computational study for rational HIV-1 non-nucleoside reverse transcriptase inhibitor selection and the discovery of novel allosteric pockets for inhibitor design

**DOI:** 10.1042/BSR20171113

**Published:** 2018-03-05

**Authors:** Ron Zhi-Hui Chiang, Samuel Ken-En Gan, Chinh Tran-To Su

**Affiliations:** 1 Bioinformatics Institute, Agency for Science, Technology, and Research (A*STAR), Singapore 138671; 2 p53 Laboratory, Agency for Science, Technology, and Research (A*STAR), Singapore 138648

**Keywords:** Allosteric pockets, cross resistance, HIV drug resistance, Non-nucleoside RT Inhibitors

## Abstract

HIV drug resistant mutations that render the current Highly Active Anti-Retroviral Therapy (HAART) cocktail drugs ineffective are increasingly reported. To study the mechanisms of these mutations in conferring drug resistance, we computationally analyzed 14 reverse transcriptase (RT) structures of HIV-1 on the following parameters: drug-binding pocket volume, allosteric effects caused by the mutations, and structural thermal stability. We constructed structural correlation-based networks of the mutant RT–drug complexes and the analyses support the use of efavirenz (EFZ) as the first-line drug, given that cross-resistance is least likely to develop from EFZ-resistant mutations. On the other hand, rilpivirine (RPV)-resistant mutations showed the highest cross-resistance to the other non-nucleoside RT inhibitors. With significant drug cross-resistance associated with the known allosteric drug-binding site, there is a need to identify new allosteric druggable sites in the structure of RT. Through computational analyses, we found such a novel druggable pocket on the HIV-1 RT structure that is comparable with the original allosteric drug site, opening the possibility to the design of new inhibitors.

## Introduction

HIV-1 drug resistance mutations rendering the Highly Active Anti-Retroviral Therapy (HAART) cocktail [1] ineffective have been increasingly reported. Currently, the HAART cocktail consists of reverse transcriptase (RT) inhibitors (RTIs), protease inhibitors, and integrase inhibitors. Together they work to interfere with virus replication, maturation, and viral genome integration, respectively [2]. Of these three enzymatic drug targets, only the RTIs have two classifications based on their modus operandi as nucleoside RT inhibitors (NRTIs) and non-nucleoside RT inhibitors (NNRTIs). The NNRTIs functions non-competitively, binding to an allosteric site to cause structural changes to the RT polymerase active site, whereas the NRTIs directly compete in the active site with nucleotides during the incorporation to terminate the reverse-transcription process [3]. Given that the NRTIs work competitively, NRTI drugs are generally nucleotide analogs, and thus limited in structure. On the other hand, the NNRTI allosteric inhibitors that distantly influence the RT polymerase active site, is open to a wider scope of ligand structures.

NNRTIs were first discovered [4,5] through multiple compound library screening [6], in which the two derivatives ‘HEPT’ and ‘TIBO’ were found to selectively inhibit HIV-2 replication *in vitro*. Many NNRTIs were later developed [7] using these two derivatives as study templates. Although the first NNRTI drug (nevirapine or NVP) was approved in 1991 [8], its RT inhibition mechanism of affecting RT flexibility was only recently reported [9]. Another NNRTI, efavirenz (EFZ), binds to the p66 subunit of HIV-RT and restricts the motions and conformational changes of the RT thumb that is necessary for DNA polymerization. In general, the NNRTIs distort the polymerase primer grip, thereby inhibiting the proper positioning of the primer at the 3′-end polymerase active site [10]. Due to the distal effects, the mechanism is deemed to be allostery [9,11]. Further using hydrogen exchange MS, Seckler et al. [9] revealed an allosteric network in EFZ-bound RT structure involving both RT subunits p66 and p51, demonstrating that p51 also underwent substantial conformational changes in addition to p66 in order to trigger allosteric couplings upon NNRTI binding.

To overcome NNRTIs, the viral RT was found to gain mutations that changed the physicochemical properties of the drug-binding pocket [12,13] and/or to disrupt the allosteric mechanism [11]. Given the limited NNRTI options in HAART, there remains a need to extend the effectiveness of the available NNRTIs by delaying the onset of cross-resistance. Further taking advantage of the allosteric coupling, there is a need to identify new druggable pockets, to which future drugs can be designed to synergistically inhibit RT function.

Our paper aims to address these two goals by using the latest reported clinical NNRTI resistant mutations [14] for integration into RT structures as study models. We sought to study the NNRTI drug-resistance mutations in cross-resistance by analyzing structural correlation-based networks of the mutant RT–NNRTI complex structures. In addition, we searched for additional allosteric pockets that influenced the polymerase active site that are comparable with the known NNRTI allosteric pocket. The identification of such new pocket(s) is important for the advent of new NNRTIs, particularly in the context of WHO guidelines on NNRTI drug resistance [15,16].

## Materials and methods

### Modeling the structures of RT mutants

Five wild-type RT structures: 3T19 (with M05), 1IKW (with EFZ), 3M8P (with ETV), 3HVT (with NVP), and 4G1Q (with rilpivirine or RPV)) were obtained from the Protein Data Bank (PDB) based on their co-crystallization with the above-mentioned NNRTIs. Amongst them, 3T19 was used as a control RT wild-type as it bound to the non-HAART drug M05. After missing residues were added using MODELLER [17], computational mutagenesis was performed on these RT-NNRTI complexes with the 2017 clinically reported NNRTI resistance mutations [14] using SCWRL4 [18], which successfully reproduced the side chain of the control RT structure (with RMSD ~0 Å).

A total of 14 mutant complexes (shown in Supplementary Table S1) and 4 wild-type (complexed with respective NNRTIs) structures were then subjected to energy minimization to remove steric clashes using Gromacs [19] version 5.1.4 with the Gromos 43a1 force field. A cubic solvent box (spc216) was used with the protein centered at 1.0 nm from the box edges. Chlorine ions were added to obtain a neutralized net charge for the whole protein system, and a standard procedure [19] of energy minimization was performed.

### Establishing the structural correlation network

Using Gephi v0.8.1 [20], a weighted directed graph *G* = (*V, E*) was constructed with nodes *V* connected by edges *E*. The nodes represent the 14 mutant RT–NNRTI complex structures and the edges represent the pairwise structural relations between the nodes. Each individual edge was characterized by quantitating the structural correlation using Pearson correlation *R*(*C_vi_, C_vj_*), where *C_vi_ and C_vj_* are two normalized parameter characterized vectors assigned for nodes *i* and *j*, respectively. The vector *C_v_* includes: (i) NNRTI-binding pocket volume, (ii) allosteric communications between mutational sites and the DNA-binding pocket (i.e. polymerase active site), (iii) thermal stability caused by the mutations, and (iv) structural deviation caused by the mutations. Each vector was defined as below:

- *NNRTIs binding pocket volume*: Using ‘dpocket-pocket descriptor extraction’ in the protein cavity detection package *fpocket* [21], the drug-binding pocket volume was estimated for each modeled RT–NNRTI mutant complex structure. Default parameters were used.
- *Allosteric communication of the mutations to the DNA-binding pocket (polymerase site):* The energy minimized mutant RT–NNRTI’s structures were submitted to the Server for Allosteric Communication and Effects of Regulations (SPACER) [22] to estimate the allosteric communication between the reported mutations (Supplementary Table S2) and the DNA-binding pocket. The allosteric communication was quantitated via the ‘leverage coupling’ concept (refer to Goncearenco et al. [22] for more details) in SPACER.
- *Structural thermal stability:* Thermal stability of the modeled RT–NNRTI complex structures were evaluated using the ENCoM [23] (standalone version; according to the protocol [24]) with the wild-type control (PDB: 3T19) for the corresponding mutations. The estimated free energy change (ΔΔG including vibrational entropy and approximated enthalpy scores) representing the thermal stability was calculated by linearly adding all the individual energy scores of all residues.
- *Structural deviation:* The RMSD was calculated to take into account the structural deviation caused by the different drug-resistance mutations. This was performed by structural alignment of the minimized mutant structures against the control wild-type (PDB: 3T19) using PyMol (https://pymol.org).

A consolidated cross-resistance map was generated to reflect dominant directions between the main representing nodes (i.e. NNRTIs). In this map, the directed links were weighted using the ratio of total weighted connections of each NNRTIs over the total number of links (i.e. *n*=36) shown in the network.

### Prediction of novel allosteric pockets in RT

The prediction of novel allosteric pockets on the wild-type RT (PDB:3T19) was performed using the *fpocket* program [21]. We first evaluated the reliability of the *fpocket* prediction on its identification of the known NNRTI-binding pocket, which was ranked second overall and had the highest ‘druggability’ score in the top five identified pockets (see Supplementary Figure S1). We then independently performed allosteric pocket prediction for PDB:3T19 on the AlloPred server [25] (refer to Greener and Sternberg [25] for more details), and found that four out of five identified pockets above were predicted to be ‘allosteric’ (with the known NNRTI-binding pocket as the highest ranking allosteric pocket). Hence, we considered the other three following ranked pockets as possible novel allosteric pockets.

To quantitate the allosteric effects on to the DNA polymerase active site by the predicted allosteric pockets, we applied normal mode-based approach to consider the distal effects between the two large subunits of RT (i.e. effects caused by the pockets on the p51 subunit to the polymerase active site on the p66 subunit). For this, we used a statistical mechanical model [26] (implemented in the AlloSigMA server [27]) to estimate the energies exerted by the allosteric communication.

In the AlloSigMA server, the allosteric communications were estimated based on the responses of each residue (via the calculated free energy ΔG_residue_) with respect to perturbations due to binding events [27]. Hence in this analysis, we first simulated the binding of small molecules at these predicted pockets P1, P2, and P3 (residue regions shown in Supplementary Table S3) by initiating the perturbations. The resulting residue-wise allosteric free energies (ΔG_residue_ with negative values indicating stabilizing and positive values indicating destabilizing effects) showed the allosteric responses at each position caused by the simulated binding events. Next, we calculated free energy changes (ΔG_site_) of both the polymerase active site and NNRTI-binding pocket by linearly adding all the energies (ΔG_residue_) of the involved residues constituting the site/pocket with respect to the independent perturbations at the three identified pockets. For statistical analysis, we used various wild-type RT structures (3T19, 1IKW, 3M8P, 3HVT, and 4G1Q) as repeats for the energetics estimations of the three identified pockets.

As an added control, we simulated DNA binding or NNRTI binding at the polymerase active site and the known drug-binding site as perturbations, respectively, using AlloSigMA server in the same manner to identify a four-residue patch (located in the subunit p51) that was least allosterically affected (ΔG_residue_ ~0). This four-residue patch was used as the negative control site for comparisons.

## Results and discussion

### Structural relationships of NNRTI cross-resistance

We set out to investigate the structural mechanisms underlying NNRTI cross-resistance as was previously performed for HIV-1 protease [28]. In doing so, we computationally analyzed structural parameters of the 14 mutant and wild-type RT structures such as the pocket volumes of the NNRTI-binding pocket, allosteric communications between the mutational sites and the DNA polymerase site, and the overall thermal stability of the RT–NNRTI complex structures.

We first estimated the NNRTI-binding pocket volumes of all the complex structures of RT–NNRTIs (EFZ, etravirine (ETV), NVP, and RPV) using *fpocket* [21]. This was necessary as the NNRTI-binding pocket could elastically adapt to the size of the NNRTIs, e.g. the pocket enlarged for larger inhibitors such as ETV and RPV (Supplementary Table S1).

To investigate the the allosteric communications elicited by the mutations [14], we used SPACER [22] and found that the mutations structurally influenced the polymerase active site, suggesting that the restriction of structural motions [9] may underlie RT inhibition. The drug-resistant mutants ETV4, RPV4, and RPV5 reflected the most drastic changes (Supplementary Table S1). These reported drug-resistant mutants contain the single p51-mutation E138Q/R at the rim of the NNRTI-pocket entrance. The E138 mutation was found to decrease allosteric communication to the polymerase site in ETV4 (as E138Q) but increased allosteric communication in RPV4 and in RPV5 (as E138R). This was also observed when E138 mutations occurred together with other mutations in RPV1, in ETV1, or in ETV2 to synergistically increase allosteric communication. The estimated free energy change (ΔΔG) using the ENCoM [23] revealed that this single mutation E138Q/R on the p51 subunit contributed to stabilize the overall RT structure (with negative ΔΔG in cases of mutants ETV4, RPV4, RPV5 shown in Supplementary Table S1).

To evaluate the effects of the drug-resistance mutations on overall structural stability, as performed in our previous studies [28,29], we found a trend that the mutations generally destabilized the RT structures (positive ΔΔG in Supplementary Table S1) with the exceptions ETV4, NVP2, RPV3, RPV4, RPV5. Most notable amongst the destabilizing mutations is Y181 found in all the NNRTI resistance sequences (with Y181C showing a greater destabilizing effect than Y181I). However, it should be noted that unlike protease, RT, particularly its p66 subunit, requires flexibility [30] for functioning. This suggests that these mutations conferred additional flexibility for RT to adopt different conformations in the presence of the inhibitors. We observed structural changes arising from these mutations where the 14 RMSD values of the mutants ranged from 0.7 to 2.4 Å when structurally aligned to the control (PDB: 3T19). We incorporated these differences into the integrated vector *C*_v_ to include structural contribution effect and avoid possible biases toward allosteric communication (*AllosCom*) and free energy change (ΔΔG) during the network construction. Nonetheless, we are aware that these RMSD values may not fully reflect the local dynamics caused by the mutations.

**Figure 1:**
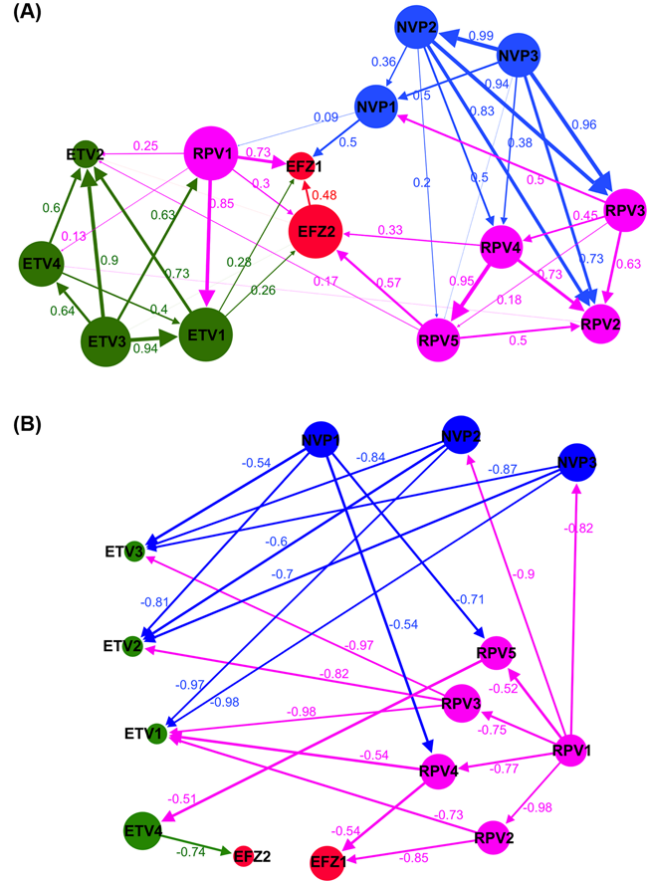
Structural correlation network of the cross-resistance relationship amongst the RT–NNRTI complexes (**A**) The positive correlation network between the NNRTI resistance of the 14 mutant complexes of RT and NNRTIs: EFZ, NVP, ETV, and RPV. (**B**) The inverse correlation network between the NNRTI resistance mutations. Labels are represented the same as in (A). In both (A,B), the complexes are numbered according to the mutated sequences shown in Supplementary Table S3. The calculated Pearson correlations are shown as edge weights. For simplicity in (B), only connections with R~[−1, −0.5] are shown. The edges are colored with respect to the source nodes, representing the outward links from one node to the others. Node size (measurement based on ‘closeness centrality’) presents how closely one node is connected to the others.

The structural correlation-based networks (Figure 1) were constructed using the characterized vectors (tabulated in Supplementary Table S1). Edge weights were assigned using the Pearson correlation (*R* ∈ [−1.0, 1.0]) between the normalized vectors. The positive *R* correlation network of the NNRTI complex mutants (Figure 1A) shows that most of the mutants (i.e. nodes in the network) that conferred resistance to the same NNRTI are highly correlated (shown as thick arrows in Figure 1A). Given that each mutant bears varied mutation combinations (refer Supplementary Table S3), there are several key positions shared between the mutants that can determine the structural characteristics to resist particular NNRTIs, e.g. NVP2 and NVP3 (*R* = 0.99), ETV3 and ETV2 (R = 0.9), or ETV3 and ETV1 (*R* = 0.94). Across different NNRTIs, high correlations amongst NVP3–RPV3, NVP2–RPV3, NVP2–RPV2, RPV1–ETV1, and RPV1–EFZ1 suggest cross-resistance between these NNRTIs.

Interestingly, the EFZ-resistant mutations (i.e. EFZ1 and EFZ2) were observed to have low correlations (less than 0.5) between each other. Comparison of the structural characteristics (Supplementary Table S1) showed distinct differences in the estimated free energy changes (ΔΔG) caused by the mutations between EFZ1 and EFZ2, in particular with significant changes in solvent exposure at the two drug-binding positions Y181 and G190 (e.g. polar Y181C-G190S in EFZ1 compared with hydrophobicity in Y181I-G190A in EFZ2). Unexpectedly, EFZ1 and EFZ2 were observed to have no significant links to the other NNRTI-resisting mutants (Figure 1A), suggesting that patients with these mutations can still be treated with other NNRTIs without rapid emergence of specific drug-resistant HIV variants.

In the inverse correlation graph (Figure 1B with negative correlations *R* ∈ [−1.0, −0.5]), we noticed that RPV and ETV resistance serve as bridges between NVP and EFZ resistance. These mutations appear to be inversely correlated to NVP and EFZ mutations. This suggests that certain mutations within one drug resistance can suppress the emergence of negatively correlated mutations to other drugs. The co-administration of two NNRTIs may then force RT toward a bottleneck, i.e. the use of NVP and RPV to select for NVP- and RPV-resistant variants would strongly suppress the emergence of ETV-resistant mutations.

**Figure 2:**
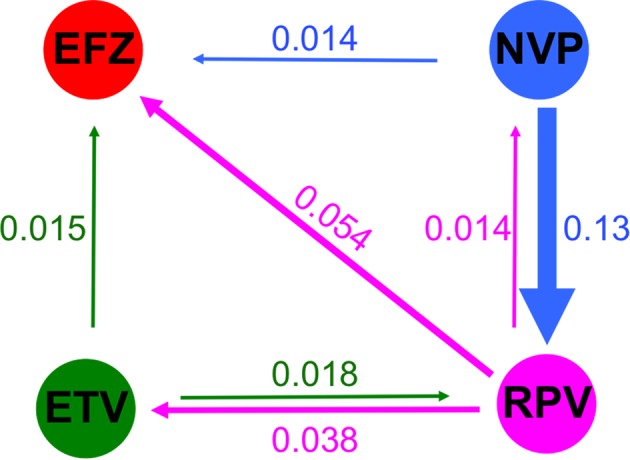
Consolidated guidance map for the usage of four respective NNRTI drugs The directed and colored arrows indicate the likelihood of cross-resistance from the respective drugs. The links were weighted using the ratio of total weighted connections of each NNRTIs over the total number of links (i.e. *n*=36) shown in the positive correlation network. For example, between NVP and EFZ, (1 × 0.5)/36 = 0.014.

A consolidated network (Figure 2) derived from the positive network (Figure 1A) was further plotted to reflect the dominant drug resistance trend (shown as weighted arrows) across the NNRTIs based on the calculated ratio scores (see ‘Materials and methods’ section). A convergence of cross-resistance toward EFZ was observed. Notably, NVP resistance showed the highest likelihood of developing cross-resistance toward RPV (ratio of 0.13), with approximately ~3–10 folds higher cross-resistance when compared with the other drugs. Further support for the use of EFZ as a first-line drug could be implied from the finding [31] that EFZ, a second-generation NNRTI, is more flexible thus allowing it to reposition better in the binding pocket despite conformational changes, thereby being more resilient to drug resistance.

On the other hand, our correlation map in Figure 1A suggests that RPV should be avoided as the first line of treatment, but rather be used as the last line, given that the drug tends to be a hub leading to cross-resistance to other NNRTIs. Our weighted ratio (in Figure 2) showed that RPV cross-resistance was higher toward EFZ at ~0.054, then ETV (~0.038) and finally to NVP (~0.014). Our model was supported by a previous study [32] where the mutation E138K was found to be prominent in RPV resistance, and it alone was sufficient to result in drug resistance. E138 mutations were also found most amongst RPV-resistant sequences than for the other drugs [14].

Further analysis of the EFZ1 and RPV1 (Figure 1A) suggest NVP to be a suitable second-line drug for patients carrying RPV1-resistant mutations (refer to Supplementary Table S2). While RPV mutants generally demonstrated high correlations with other NVP mutants, RPV1 mutations are negatively correlated to NVP2- and NVP3-resistant mutations (Figure 1). Different mutations (e.g. at K103 and V106 in NVP2 near the drug-binding site, H221 and F227 in RPV1 near the polymerase active site, and different Y181 mutations) might have caused the varied allosteric communications and structural stability (shown in Supplementary Table S1), leading to the specific resistance to the drugs. These observations suggest that those key positions play a role in influencing the usage of these EFZ and RPV.

### Search for novel non-competitive druggable sites

We were able to gain valuable insights and a potential treatment guide for the use of the various NNRTIs from the network. Compared with protease inhibitors, fewer NNRTIs are used clinically and thus higher cross-resistance amongst the NNRTIs is expected. To overcome this, there is a clear need for novel druggable allosteric pockets to be identified in HIV-RT for the development of new generation NNRTIs.

**Figure 3:**
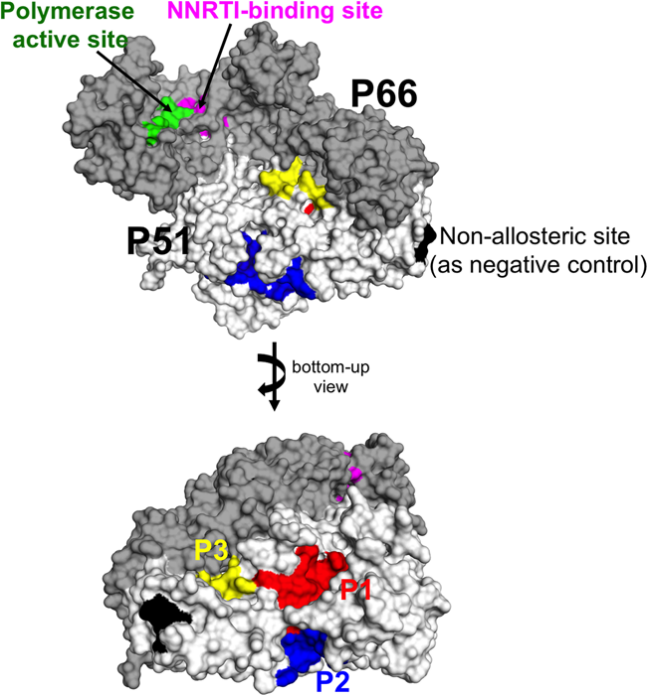
Three predicted allosteric pockets The RT structure is shown in surface presentation (p66 in dark gray and p51 in white), the three pockets P1 in red, P2 in blue, P3 in yellow, the known NNRTI-binding pocket in magenta, and the polymerase active site in green. The negative control non-allosteric site is shown in black.

Using AlloPred server, we identified three additional pocket candidates as potential allosteric pockets apart from the known NNRTI-binding site (see ‘ Materials and methods ’ section). We named these three pockets as P1, P2, and P3 according to the AlloPred ranks. While the known NNRTI-binding site is located on the p66 subunit, the novel allosteric pockets P1, P2, and P3 were identified on the p51 subunit (Figure 3 and Supplementary Table S3) and exhibited the top highest prediction scores based on their physicochemical properties (Supplementary Figure S1). All the three pockets are larger yet less hydrophobic than the known NNRTI site.

To quantitate the allosteric effect, we used the AlloSigMA server [27] to estimate the free energy changes in both the polymerase active site and NNRTI-binding pocket with respect to independent perturbations at the three pockets (see ‘Materials and methods’ section). The known NNRTI-binding pocket (magenta in Figure 3) was used as a positive control for the comparison of allosteric communications. Results of the average free energy changes (ΔG_site_ in Table 1) with respect to various sites from the five various wild-type RT structures showed that the three pockets exhibited different allosteric effects toward the polymerase active site (with P2 showing more similarities to the known NNRTI-binding pocket when compared with the other two). Like the known NNRTI-binding pocket, P2 displayed a strong stabilizing (or rigidifying) effect on the polymerase active site (ΔG_site_ <0). The other two pockets P1 and P3 exhibited destabilizing effects on the polymerase active site (with P3 < P1). Since rigidity is desired to inhibit RT functioning [33], P2 (rather than the other two) is a more suitable allosteric pocket candidate.

**Table 1:** Average free energy changes (ΔG) quantitating the allosteric effects

Sites/pockets^1^ (where perturbed, to simulate ligand binding)	ΔG_site_ (kcal/mol) of corresponding pockets/domains			
	Polymerase active site	NNRTI-binding pocket	p51^2^	p66^2^
Predicted pocket P1	0.83 ± 1.39	−6.59 ± 1.37	−141 ± 72	111 ± 60
Predicted pocket P2	**−**9.36 ± 0.92	−28.2 ± 3.47	−204 ± 47	100 ± 67
Predicted pocket P3	0.23 ± 2.12	−0.59 ± 3.16	−150 ± 103	47 ± 47
**Known NNRTI-binding pocket**	**−2.68 ± 1.36**	**—**	**−79 ± 39**	**24 ± 25**
Non-allosteric site	0.45 ± 0.58	1.83 ± 0.97	−4.3 ± 9.65	36 ± 65

The known NNRTI-binding pocket (in bold) was used as a positive control.

1 Residues involved in these sites/pocket analyses are tabulated in Supplementary Table S3.

2 In cases of p66 and p51, the values are too large for decimals to be accounted.

To further support that P2 could allosterically stabilize the DNA polymerase active site, we performed single-point mutagenesis for each residue of the pocket P2 and detected the corresponding effects to the DNA polymerase active site (using the AlloSigMA server with the perturbations to simulate the residual mutation; details in Guarnera et al. [27] and found that most single mutations in pocket P2 induced flexibility (ΔG_site_ > 0 as shown in Supplementary Table S4) onto the DNA polymerase active site.

All the four pockets (i.e. the known NNRTI drug-binding site, P1, P2, and P3) were shown to stabilize the overall p51 subunit but destabilize the p66 subunit, from which P2 exhibited the strongest effect compared with the others (Table 1). While the interaction of p51 and the tRNA primer [34] may mediate stability to p66, P2 on p51 subunit could affect the polymerase active site on the p66 subunit. Hence, given the similar resulting effects between P2 and the known NNRTI-binding allosteric site, P2 is our most promising novel druggable pocket, especially considering that there have not yet been any NNRTIs developed to target the p51 subunit.

## Conclusion

Since there are limited implementations of the alternative non-NNRTI-based regimen as first line for treatment to some subgroup populations due to cost [15,16], the WHO guidelines do not discourage the continuing use of NNRTIs and this drug class remains a treatment, to which drug resistance ought to be addressed. Thus our work of guiding drug selection as well as providing novel potential drug sites would add to the solution currently faced for NNRTI resistance.

We constructed RT–NNRTIs cross-resistance networks that serve as a guide to NNRTI selection for HIV-1 RT to delay drug cross-resistance onset and our results support using EFZ as the first line of treatment, in agreement with the reported WHO global guidelines [16] as it has minimal cross-resistance toward other NNRTIs in HIV-1 RT. Depending on the emergence of the RT mutations, certain NNRTIs may be more useful in subsequent secondary treatments.

As an error-prone HIV-1 RT may eventually escape the four available NNRTIs, our further research found a potential candidate pocket (i.e. P2) in the p51 subunit that showed promise as a new druggable site, to which new inhibitors can be designed against HIV-1 [35].

## Supporting information

**Figure S1:** Physicochemical properties of the predicted pockets (gray), in particular P1, P2, P3, and the known NNRTI-binding pocket highlighted in red, blue, yellow, and magenta, respectively.

**Table S1:** Structural characterised factors used to construct the structural correlation-based network.

**Table S2:** Clinically identified mutation in Reverse Transcriptase from the 2015 updated HIV-1 report.

**Table S3:** Involved residues in the pockets used for allosteric communication estimation.

**Table S4:** Free allosteric energy change (ΔΔG_site_) of the DNA polymerase active site when mutating each residue of the pocket P2. The control wild type RT was used in this analysis.

## Abbreviations

EFZ: efavirenz
ETV: etravirine
HAART: Highly Active Anti-Retroviral Therapy
NNRTI: non-nucleoside reverse transcriptase inhibitor
NRTI: nucleoside reverse transcriptase inhibitor
NVP: nevirapine
PDB: Protein Data Bank
RPV: rilpivirine
RT: reverse transcriptase
RTI: RT inhibitor
SPACER: Server for Allosteric Communication and Effects of Regulations
