# Supplementary material for: A computational study for rational HIV-1 non-nucleoside reverse transcriptase inhibitor selection and the discovery of novel allosteric pockets for inhibitor design
Source: Biosci Rep. 2018 Mar 5;38(2):BSR20171113. doi: 10.1042/BSR20171113 (PMC5835713; doi:10.1042/BSR20171113)
Supplement: Supplementary file 1 [file bsr20171113_Supp1.pdf]

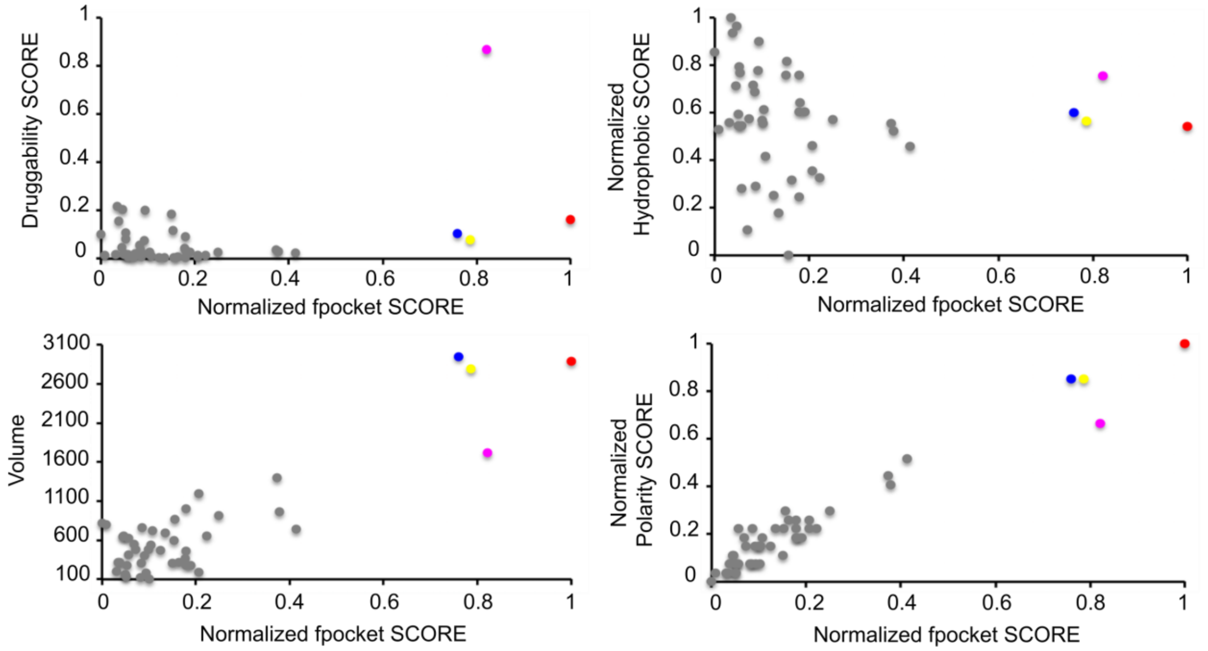

Figure S1. Physicochemical properties of the predicted pockets (gray), in particular P1, P2, P3, and the known NNRTI-binding pocket highlighted in red, blue, yellow, and magenta, respectively.

Table S1. Structural characterised factors used to construct the structural correlation-based network.

	**Vpocket (Å^3)**	**#AllosComm** (wt / negCtrl)	**##ΔΔG/ΔS (kcal/mol)**	**RMSD (Å)**
**Efavirenz (EFZ)** - Molecular Weight = ~315 g/mol				
EFZ0 (**wt**)	649.4	-	-	-
EFZ1	625.6	0.7 (0.76 / 0.14)	6.24 / 0.03	0.85
EFZ2	640.4	0.84 (0.76 / 0.14)	1.94 / 0.03	0.85
**Etravirine (ETV)** - Molecular Weight = ~435 g/mol				
ETV0 (**wt**)	930.8	-	-	-
ETV1	1155.3	0.89 (0.82 / 0.37)	7.33 / 0.06	0.73
ETV2	1266.7	0.89 (0.81 / 0.37)	1.44 / ~0	0.73
ETV3	1047.5	0.77 (0.77 / 0.37)	3.17 / 0.06	0.73
ETV4	989.6	0.57 (0.74 / 0.37)	-0.006 / -0.004	0.73
**Nevirapine (NVP)** -Molecular Weight =~266 g/mol				
NVP0 (**wt**)	636.9	-	-	-
NVP1	757.6	0.52 (0.59 / 0.07)	10.27 / 0.1	2.42
NVP2	761.0	0.63 (0.59 / 0.07)	-0.64 / 0.02	2.42
NVP3	762.3	0.62 (0.66 / 0.07)	0.64 / 0.001	2.41
**Rilpivirine (RPV)** - Molecular Weight =~366g/mol				
RPV0 (**wt**)	744.0	-	-	-

RPV1	1062.1	0.87 (0.7 / 0.1)	7.73 / 0.09	1.72
RPV2	913.4	0.76 (0.78 / 0.1)	2.0 / 0.08	1.72
RPV3	762.7	0.69 (0.74 / 0.1)	-0.05 / -0.004	1.72
RPV4	819.2	0.81 (0.68 / 0.1)	-0.18 / 0.008	1.72
RPV5	833.5	0.91 (0.68 / 0.1)	-0.18 / 0.008	1.72

[#]For comparison purposes, estimated allosteric communication values of the control wild type RT (with negative control site) are shown in parentheses

[##]$\Delta\Delta G$ and ΔS scores represent free energy and vibrational energy respectively, demonstrating thermo stability of the RT when mutated from the control wild type.

Table S2. Clinically identified mutation in Reverse Transcriptase from the 2015 updated HIV-1 report.

	V90	A98	L100	K101	K103	V106	V108	E138	V179	Y181	Y188	G190	H2221	P225	M230
**EFZ1**			I	P	N	M	I			C	L	S		H	L
**EFZ2**					S					I		A			
**ETV1**	I	G	I	E		I		A	D	C		S			L
**ETV2**				H				G	F	I		A			
**ETV3**				P				K	T	V					
**ETV4**								Q							
**NVP1**			I	P	N	A	I			C	C	A			L
**NVP2**					S	M				I	L				
**NVP3**											H				
**RPV1**			I	E				A	L	C	L		Y	C	I
**RPV2**				P				G		I					L
**RPV3**								K		V					
**RPV4**								Q							
**RPV5**								R							

Table S3. Involved residues in the pockets used for allosteric communication estimation.

	Pocket Residue
Predicted Pocket P1 (p51)	I328, Y338, K348, G352, K353, Y354, E370, A371, Q373, K374, I375, T377, E378, Q407, A408
Predicted Pocket P2 (p51)	L92, G93, I94, H96, A98, K101, G152, K154, P157, A158, Y181, Q182, Y183, M184, Y318, Y319, L349, I382, W383, G384, K385, T386
Predicted Pocket P3 (p51)	K259, I393, Q394, K395, F416, N418, T419, P420, P421, K424, L425, Q428
Drug-binding Site (p66)	P95, L100, K103, V108, Y181, Y188, G190, W229, L234, H235, Y318
DNA-binding pocket (including polymerase active site on p66)	D110, D185, D186, D76, Q151, G152
Negative control site (p51)	I293, P294, L295, T296

Table S4. Free allosteric energy change ($\Delta\Delta G_{site}$) of the DNA polymerase active site when mutating each residue of the pocket P2. The control wild type RT was used in this analysis.

Pocket P2 Residue	$\Delta\Delta G_{site}$	Pocket P2 Residue	$\Delta\Delta G_{site}$
L92	-0.18	Q182	0.57
G93	0.48	Y183	0.74
I94	0.36	M184	0.63
H96	0.36	Y318	-0.45
A98	-0.21	Y319	-0.09
**K101**	**-0.93***	L349	-0.23
G152	0.47	I382	0.18
K154	0.42	W383	0.18
P157	0.31	G384	0.14
A158	0.29	K385	0.21
Y181	0.46	T386	0.59

*some mutations, especially at the residue K101, might keep the DNA polymerase site remain rigidified, hence negating them as hot spots in the pocket P2.
